# Single Domain Antibody Multimers Confer Protection against Rabies Infection

**DOI:** 10.1371/journal.pone.0071383

**Published:** 2013-08-20

**Authors:** Bhargavi M. Boruah, Dawei Liu, Duan Ye, Tie-jun Gu, Chun-lai Jiang, Mingsheng Qu, Edward Wright, Wei Wang, Wen He, Changzhen Liu, Bin Gao

**Affiliations:** 1 CAS Key Laboratory for Pathogenic Microbiology and Immunology (CASPMI), Institute of Microbiology, Chinese Academy of Sciences, Beijing, China; 2 University of Chinese Academy of Sciences, Beijing, China; 3 National Engineering Laboratory for AIDS Vaccine, College of Life Science, Jilin University, Changchun, China; 4 Viral Pseudotype Unit, School of Life Sciences, University of Westminster, London, United Kingdom; 5 Biochemistry Teaching and Research Office, Hebei Medical University, Shijiazhuang, China; 6 China-Japan Joint Laboratory of Molecular Immunology and Microbiology, Institute of Microbiology, Chinese Academy of Sciences, Beijing, China; Public Health Agency of Canada, Canada

## Abstract

Post-exposure prophylactic (PEP) neutralizing antibodies against Rabies are the most effective way to prevent infection-related fatality. The outer envelope glycoprotein of the Rabies virus (RABV) is the most significant surface antigen for generating virus-neutralizing antibodies. The small size and uncompromised functional specificity of single domain antibodies (sdAbs) can be exploited in the fields of experimental therapeutic applications for infectious diseases through formatting flexibilities to increase their avidity towards target antigens. In this study, we used phage display technique to select and identify sdAbs that were specific for the RABV glycoprotein from a naïve llama-derived antibody library. To increase their neutralizing potencies, the sdAbs were fused with a coiled-coil peptide derived from the human cartilage oligomeric matrix protein (COMP48) to form homogenous pentavalent multimers, known as combodies. Compared to monovalent sdAbs, the combodies, namely 26424 and 26434, exhibited high avidity and were able to neutralize 85-fold higher input of RABV (CVS-11 strain) pseudotypes *in vitro*, as a result of multimerization, while retaining their specificities for target antigen. 26424 and 26434 were capable of neutralizing CVS-11 pseudotypes *in vitro* by 90–95% as compared to human rabies immunoglobulin (HRIG), currently used for PEP in Rabies. The multimeric sdAbs were also demonstrated to be partially protective for mice that were infected with lethal doses of rabies virus *in vivo*. The results demonstrate that the combodies could be valuable tools in understanding viral mechanisms, diagnosis and possible anti-viral candidate for RABV infection.

## Introduction

Rabies virus (RABV), a member of the *Rhabdoviridae* family, is a bullet-shaped virus with a non-segmented, negative-sense, single-stranded RNA genome of approximately 11 kb that encodes the following five proteins: nucleocapsid protein (N), phosphoprotein (P), matrix protein (M), glycoprotein (G), and the large subunit (L) of the RNA-dependent RNA polymerase protein (RdRp) [1]. The glycoprotein (G protein) or the envelope protein is crucial for the adsorption of RABV to the cognate host cellular receptor, which induces endocytosis of the virion. In the endosome, the acidic pH induces conformational changes in the trimeric G protein, which triggers fusion between the virus and the cell membrane [2], [3], [4]. *In vitro* studies have shown that the muscular form of the nicotinic acetylcholine receptor (nAChR) [5], [6], and the neuronal cell adhesion molecule (NCAM) [7] bind to the G protein, thereby facilitating RABV entry into cells. Although the p75 neurotrophin receptor (p75NTR) was previously reported to be a ligand for the soluble form of the RABV-G protein [8], the role of p75NTR as a RABV receptor remains obscure, as it was later reported that p75NTR is not required for RABV infection of primary neurons [9].

The mature G protein consists of the following three main moieties: the extracellular domain (20–459 aa), the transmembrane region (460–480 aa) and the cytoplasmic domain (481–524 aa). The extracellular domain is the only region in the G protein that interacts with the host cell receptor, thereby facilitating viral entry. The G protein is also considered to be the primary surface antigen that is capable of inducing and reacting with virus-neutralizing antibodies [10]. Therefore, the design of most human and veterinary vaccines is based on the functional aspects of this protein. Current rabies post-exposure prophylaxis (PEP) includes the combined administration of the rabies vaccine and the rabies immunoglobulin (RIG), the latter of which is derived from the pooled sera of either horses (ERIG) or humans (HRIG) that have been immunized using the rabies vaccine. However, PEP is reportedly ineffective upon the manifestation of the first non-specific symptoms. Additionally, factors including health risks associated with blood-derived RIG, batch-to-batch variations, and safety concerns related to blood-derived products, as well as the issue of limited supply to endemic areas, highlight the need for cheaper and more effective approaches for PEP against rabies virus infection. Alternative approaches using human monoclonal antibodies (mAbs) from transgenic mice [11] and the development of human mAb cocktails [12] have been extensively studied. The identification of RABV-specific antigen-binding fragments (Fabs) from immunized humans using a phage-display library has also been reported [13].

Single-domain antibodies (sdAbs) are derived from heavy chain antibody fragments (VHHs) occurring naturally in the sera of *Camelidae* and other dromedaries and have proven to be effective viral neutralizers [14], [15], [16], [17]. Moreover, sdAbs possess several advantages, including efficiency of expression and purification in *E. coli*, thermal stability [18], high refolding capacity [19] and efficient tissue penetration *in vivo* [20]. sdAbs can be readily used in various formats by fusion to other proteins or peptides [21]. These properties make them excellent modalities for prophylactic and therapeutic purposes. However, the small size of sdAbs (∼15 kDa) results in faster renal clearance, and the dissociation constant (Kd) of such antibody fragments (particularly from naïve or non-immune libraries) typically ranges from 10^−8^ to 10^−9^ M, making them inappropriate for several applications.

Enhancing the neutralizing potential is by far, the best way to improve the therapeutic applications of sdAbs against viral diseases, which can be achieved through several multimerization strategies. Apart from improving avidity, the multivalent format might also decrease the dissociation rates of sdAbs from target antigens and optimize their biodistribution [22]. The potency of anti-viral molecules can be further improved through exploitation of unique formatting flexibilities of sdAbs or VHHs by either fusing multiple copies of the same VHH gene or fusion of VHH genes that recognize different epitopes [23].

In this study, we have isolated and characterized sdAbs against RABV-G protein from a naïve (non-immune) llama library through phage display. To increase the binding avidity of the sdAbs, we have attempted to fuse them with a coiled-coil assembly peptide derived from the human cartilage oligomeric matrix protein (COMP48), resulting in a homogenous pentavalent structure known as combody. The alpha-helical coiled-coil peptide has been largely exploited for protein design mainly due to the efficient oligomerization and stability conferred by the complementary hydrophobic interactions between neighboring helices [24], [25], [26], [27], [28], [29]. COMP48 also aids in cell-cell adhesion by mimicking the cluster formation of E-cadherin on the cell surface [30]. The use of COMP48 has also been reported in the design for soluble inhibitors of FasL and CD40L [31]. The applicability of COMP48 to generate potent sdAb multimers has been extensively demonstrated in our laboratory [32]. We have selected and expressed a number of sdAbs specific for the RABV-G protein and have chosen two pentamers or combodies, namely 26424 and 26434, as potent neutralizing multimeric sdAbs. BR 2.3, a sdAb isolated from the same naive llama through phage display, has been used as a control sdAb in the monomer format. Both the monomer and combodies could be expressed homogeneously using a prokaryotic cell expression system. These data can help to demonstrate the applicability of sdAb multimers as potent anti-viral molecules for the diagnosis and therapy of viral infections.

## Methods

### Ethics Statement

All animal experiments were performed in strict accordance with the animal ethics guidelines recommended by the Chinese Academy of Sciences (CAS). The protocol was approved by the ethical committee of the Institute of Microbiology, CAS (Permit Number: CASPMI 012). All measures were taken to minimize sufferings of the animals and sacrifices were made at humane endpoint. The mice were examined daily for definitive clinical signs of rabies infection and were euthanized in extreme conditions by CO_2_ intoxication. The experiment was carried out for a total of 28 days post rabies virus inoculation, after which the survivors were similarly euthanized.

### Cells and viruses

Human embryonic kidney-293T cells (HEK-293T; ATCC CRL-11268) and baby hamster kidney-21 clone 13 cells (BHK-21; ATCC CCL-10) were grown at 37°C and 5% CO_2_ in DMEM-10 (Dulbecco's Minimal Essential Medium (Gibco) supplemented with 10% fetal calf serum (FCS) and 100 μg ml^−1^ streptomycin). The RABV, aG strain [33], was used as the antigen in enzyme-linked immunosorbent assays (ELISAs) during the bio-panning process for binder selection as well as for purified sdAb ELISA. The virus was inactivated using 0.05% (v/v) β-propiolactone to eliminate viral infectivity completely while maintaining antigenicity [34].

### Isolation of positive clones specific to RABV-G protein

The repertoire of sdAbs was isolated from a naïve llama library (Wellcome Trust Sanger Institute, Cambridge, UK) by phage display technique through infection into TG1 bacteria and KM13 helper phage as previously reported [35]. The frozen antibody library was thawed on ice and diluted with 500 ml of 2×TY medium containing 100 µg ml^−1^ of ampicillin. The TY medium was supplemented with 4% (w/v) glucose for suppressing antibody expression during bacterial culture. The cultures were grown in 2-liter flasks at 37°C and 216 r.p.m. until the OD_600_ increased from an initial absorbance of ∼0.1 to approximately 0.5. KM13 helper phages were added to a concentration of 2×10^12^ phages to the bacterial culture and incubated in a water bath at 37°C for 30–60 min. The cells were recovered by centrifugation at 3,200 g for 10 min at 4°C in 50-ml Falcon tubes, and the pellets were resuspended in 500 ml of 2×TY medium containing 0.1% (w/v) glucose, 100 µg ml^−1^ of ampicillin and 50 µg ml^−1^ of kanamycin. The culture was further grown at 25°C and 216 r.p.m. for 16–20 h in a 2-liter flask. Cells were recovered by centrifugation at 3,200 g for 10 min at 4°C in Falcon tubes. The resulting supernatants were filtered through 0.45 μm filters. The phages were precipitated from the filtered supernatant by incubation in a polyethylene glycol (PEG) solution (20% PEG 6000, 2.5 M NaCl) on ice for 1 h, followed by centrifugation at 3,200 g for 30 min at 4°C in ten Falcon tubes. The pellets were resuspended in 5 ml of PBS buffer and pooled together in a 15-ml Falcon tube to which 1 ml of PEG solution was added. The suspension was further incubated on ice for 10 min and then centrifuged at 3,200 g for 30 min at 4°C. The resulting pellet was resuspended in PBS. The phage solution was diluted in 1% Casein-PBS (CPBS) and incubated for 30 min on Nunc Maxisorp plates coated with whole RABV (inactivated using 0.05% (v/v) β-propiolactone) as the antigen. The plates were washed with PBS-0.05% Tween-20 ten times, and the bound phages were eluted with 200 μl of trypsin-PBS. The eluted phages were used to infect fresh TG1 cultures (OD600∼0.5) or concentrated by PEG precipitation. After three subsequent rounds of bio-panning, 56 colonies were selected, and periplasmic extracts containing the sdAb gene were prepared according to standard protocols. Selected clones were sent for sequencing.

### Construction of sdAb monomers and combodies

The encoding sequences of the selected sdAbs were cloned into the *NcoI* and *NotI* restriction sites of the C-terminal His_6_ tag-containing pET20b vector (Novagen). The sdAb gene (monomer) was amplified using the following primers: forward, 5′-CAGCCGGCCATGGCCCAGG-3′; and reverse, 5′-ATTATTATGCGGCCGCTCAATGGTGATGGTGATGGTG-3′. The generation of the pentameric constructs (combodies) was performed by cloning the sdAb sequences into the N-terminus along with the coiled-coil domain of human COMP (Asp29-Gln76, COMP48), *myc*-epitope and polyhistidine tag into the C-terminus of the vector pET26b(+) (Novagen) [32]. The oligonucleotides 5′-TAATAAGAAGACCGCAGGCCCAGGTGCAGCTGGTGGAG-3′ and 5′-ATTATTTGGGC CCTGAAGAGACGGTGACATTGT-3′ were annealed and cloned into the *NcoI* and *NotI* sites of the vector. The respective vectors were chosen based on their suitability for obtaining periplasmic proteins from the *E.coli* strains.

### Expression and purification of the monomeric sdAbs and combodies

For the production of the soluble sdAbs, we used the *E.coli* strain BL21 Gold. The cells were grown in 5 ml LB Broth (100 μg ml^−1^ kanamycin or ampicillin) and grown at 37°C with shaking at 220 r.p.m. overnight. The cultures were diluted to 1 L or 2 L LB Broth (with 100 μg ml^−1^ kanamycin or ampicillin) at a ratio of 2∶1 and grown at 37°C until a 600 nm absorbance of between 0.5–1 was obtained. Protein expression was induced by treatment with 1 mM isopropyl-B-thio-galactoside (IPTG) at a lower temperature of 22°C with shaking at 180 r.p.m. for 20 h. Cells were pelleted at 8,000 g at 4°C for 15 min and re-suspended in 1 M PBS (pH 7.4). 5 mg of lysozyme was added and incubated for 45 min at RT. The lysed cells were sonicated using a sonic dismembrator to reduce the viscosity of the lysate and centrifuged at 12,000 r.p.m. to obtain clear supernatants containing the periplasmic protein. The His_6_-tagged proteins (BR 2.3, 26424 and 26434) were purified using Immobilized Metal ion Affinity Chromatography (IMAC) and Nickel Sepharose^TM^ Fast Flow (GE Biosciences) according to the manufacturer's protocols. The desired sdAbs were eluted using 500 mM imidazole after extensive washing with buffer containing lower concentrations of imidazole.

BR 2.3, 26424 and 26434 were further purified by size exclusion chromatography using a Sephadex 200 (GE Biosciences) column on an AKTA purifier 2000 system (GE Biosciences). The pentameric and monomeric proteins were collected at the indicated elution volumes (see Results).

### Binding specifity through ELISA

For the analysis of the specificity of monomer and the combodies, 96-well Maxisorp plates (Nunc) were coated with purified RABV (aG strain, inactivated using 0.05% (v/v) β-propiolactone) at 100-fold dilution overnight at 4°C. For negative control, influenza (H1N1) virus (PR8) with selective mutations in the PB1 and PB2 genes [36], was used as the coating antigen diluted to similar concentration. After thorough washing, the wells were blocked in MT Buffer (PBS/2% skimmed milk/2% Tween-20) for 2 h at 37°C. After three washes with PBS, optimized concentration or dilutions of the sdAbs were added to the wells and incubated for another 2 h at RT with shaking. After 5 washes with PBST (PBS/0.05% Tween-20), the binding of the sdAbs was detected using a mouse anti-*myc* mAb (Epigen) followed by secondary probing with a goat anti-mouse horseradish peroxidase (HRP) conjugate. HRP activity was determined using 3,3′,5,5′-Tetramethylbenzidine (TMB) substrate. 2 M sulphuric acid (H_2_SO_4_) was used to stop the reaction, and the readings at 450 nm wavelength were measured using an ELISA-plate reader.

### Preparation of CVS-11 pseudotypes

We used the CVS-11 strain (Challenge Virus Standard-11, ATCC reference VR-959) for viral pseudotype preparation, as this is the standard internationally recognized virulent strain for laboratory use. The CVS-11 pseudotypes were prepared as previously described [37]. For transfections, 5×10^6^ HEK-293T cells were grown in DMEM-10 (Dulbecco's Minimal Essential Medium (DMEM) supplemented with 10% fetal calf serum (FCS) and 100 μgml^−1^ streptomycin) for 24 h prior to the addition of the combination of constructs. The plasmids pLP1 (HIV *gag-pol*)and pLP2 (RSV promoter) (BLOCK-iT ™ Lentiviral RNAi Expression System, Invitrogen), which supply the helper functions as well as structural and replication proteins to produce lentivirus, were transfected together with the firefly *luciferase* reporter pCSFLW plasmid and with the CVS-11 envelope construct, pI.18-CVS-11. The supernatants containing the pseudotype viruses were harvested at 72 h post-transfection and were stored in aliquots for short-term at 4°C or for long-term at −80°C.

### In vitro analysis using pseudotype neutralization assay

In 96-well flat-bottomed plates (Corning, USA), threefold serial diluted or optimized concentration of the sdAbs (diluted in DMEM without serum or antibiotics) ranging from 0.6 μg ml^−1^ upto 50 μg ml^−1^ were incubated with 50 μl of CVS-11 pseudotype viruses (final volume of 200 μl) for 1 h at 37°C in a BOD incubator (5% CO_2_). For control experiment, Human Rabies Immunoglubulin (HRIG) (Shandong Taibang Biological Products Co. Ltd.) at similar concentrations was used as a positive control. The antibody-pseudotype mixtures were then added to 96-well plates, pre-seeded overnight with monolayer cultures of BHK-21 cells at a concentration of 5000 cells per well and incubated for 5 h at 37°C in a BOD incubator (5% CO_2_). Negative control comprised of wells containing CVS-11 pseudotypes without any antibody treatment. The medium was then replaced with DMEM containing 5% FCS (heat-inactivated at 56°C for 30 min) and incubated further for 48 h.

Luciferase reporter activity in transduced cells was quantified using the Fire-Lucy Assay Kit (Vigorous Biotechnology Beijing Co. Ltd.). The cells were washed twice in PBS (pH 7.4) and lysed with 1X Universal Lysis Buffer according to the manufacturer's protocols. The cells were detached by shaking for 5 min, and the sdAb dilution-treated lysates were collected in fresh eppendorf tubes and immediately placed in ice. For detecting luciferase activity, 20 μl of each lysate was added to individual wells of a white opaque 96-well plate, and 100 μl of luciferase substrate solution was added prior to measurement. The plate was measured using a Glomax 96 microplate luminometer (Promega), and the relative light unit (RLU) values were compared to the negative control containing CVS-11 pseudotypes only. A 50% or more reduction in RLUs of the cells was considered indicative of virus neutralization.

RLU output against each dilution of the sdAbs (26424, 26434 and BR 2.3) were plotted in a graph and the mean standard deviations (±SD) were calculated. The RLU values of candidate sdAbs were compared with that of HRIG (currently used in PEP for rabies in China), which served as a positive control for all subsequent neutralization assays.

### Assessment of the efficacy of combodies using mouse neutralization test (MNT)

Based on their efficacy to neutralize the CVS-11 pseudotypes, the combodies, 26424 and 26434 were chosen for testing their neutralizing ability in a mouse RABV challenge model, according to guidelines from World Health Organization (WHO). Rapid Fluorescent Focus Inhibition test (RFFIT) was conducted for the combodies (including ERIG) and mouse neutralization test (MNT) was performed as previously described [38], [39]. The titers of 26424 and 26434 were estimated to be 1.6 IU ml^−1^ and 0.2 IU ml^−1^. Briefly, 26424 (0.3 mg ml^−1^) and 26434 (0.6 mg ml^−1^) were individually mixed with 2500 LD_50_/5 μl RABV (CVS-24 strain) [40] in separate tubes and incubated in ice for 1 hour. The antibody-virus mixture was then injected in the hind leg of the mouse (Kunming strain, 6 mice per group, each weighing 11–13 g). The positive control consisted of ERIG (Wuhan Institute of Biological Products Co. Ltd.) at 15.4 IU ml^−1^ using a similar administration procedure. The mice were vaccinated intraperitoneally on days 0, 3, and 7 with 0.5 ml rabies vaccine (Jilin Maifeng Pharmaceutical Co. Ltd.) that was diluted at a ratio of 1∶25 (v/v) with PBS. The negative control consisted of the two following groups: one injected with PBS only and one injected with PBS and vaccine. The mice were examined daily for definitive clinical signs of rabies infection and were euthanized in extreme conditions by CO_2_ intoxication. The experiment was carried out for a total of 28 days post rabies virus inoculation, after which the survivors were similarly euthanized. Postmortem diagnosis of rabies infection by direct fluorescent antibody testing was performed on each mouse [41].

Kaplan–Meier survival curves were generated using GraphPad Prism5. The statistical analysis of the survival curves was done according to Mantel–Cox test.

## Results

### Selection of RABV-G specific sdAbs

**Figure 1:**
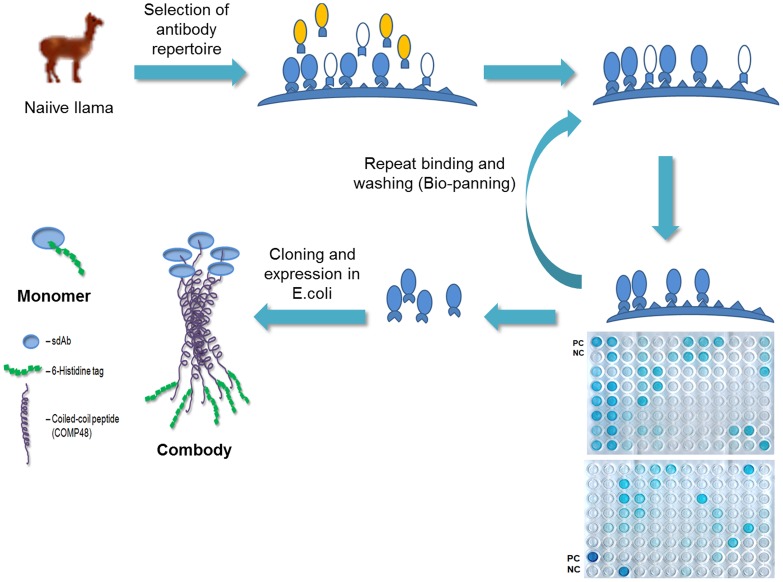
Schematic illustration of the isolation of sdAbs from naïve llama library through phage display. Whole RABV (inactivated) was used as antigen to screen the phages during bio-panning. Positive control (PC) and negative control (NC) were included in each plate during phage ELISA. The strongest positive clones were selected for subsequent cloning and expression as monomer and multimer (combody) in E.*coli*.

**Figure 2:**
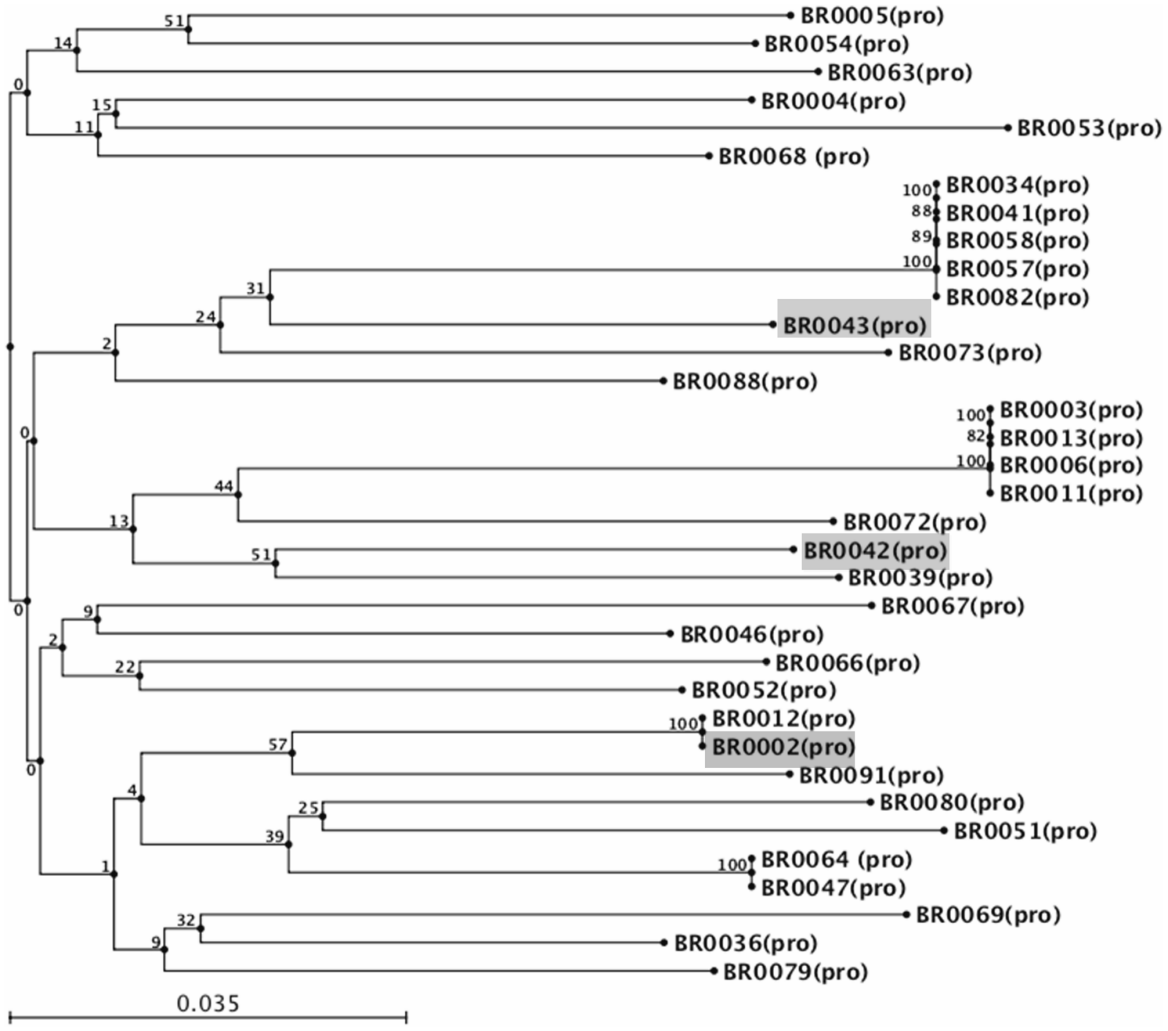
Neighbor-Joining phylogenetic analysis of the sdAb gene sequences isolated through phage display from naïve llama library. 35 strong positive clones (from a total of 1000 clones) were screened for amino acid homology in the complementary determining regions (CDRs) of the sdAb gene. Out of them, 16 sequences could be efficiently cloned and expressed in E.coli expression system. The shaded clones depict the sdAb genes investigated in our study. Combodies, 26424 and 26434, were derived from BR0042 and BR0043 respectively; whereas the monomer, BR 2.3, was derived from BR0002. The tree topology with bootstrap values for 100 replicates is constructed using CLC Sequence Viewer 6.

The sdAb genes were isolated using phage display technique from a naïve llama library and screening was performed using whole, inactivated RABV (aG strain). After extensive washing steps, bound phages were rescued or eluted though reactions with trypsin-PBS. Enrichment in specific binders was performed over three rounds of *in vitro* selection or “bio-panning”, The positive clones were further assessed for antigen specificity and binding by phage ELISA (Figure 1). A total of 35 clones were identified (out of 1,000 clones) for further screening and characterization (Figure 2).

### Preparation of the sdAbs in monomeric and multimeric formats

**Figure 3:**
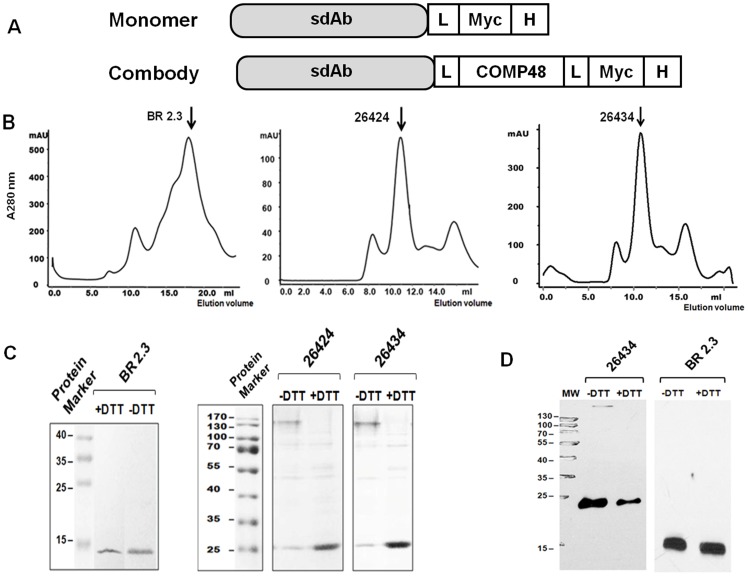
Characterization of sdAb monomer and combodies. (A). Primary structure of monomer and combodies of the sdAbs used in our study are shown. (B) Size-exclusion chromatography of BR 2.3, 26424 and 26434. The size of the monomeric and multimeric sdAbs was analyzed through Sephadex 200 chromatography and the elution positions have been depicted. (C) The size of BR 2.3, 26424 and 26434 has been further confirmed through SDS-PAGE. BR 2.3 elutes as a 14 kDa monomer in both reducing (+DTT) and non-reducing (-DTT) conditions. Combodies 26424 and 26434 elutes as 25 kDa protein in reducing conditions and appears to be more than 130 kDa in non-reducing SDS-PAGE, suggesting pentamerization of the coiled-coil peptide. (D) The monomeric and pentameric sdAbs were further analyzed in Western blot. The purified proteins were run in a 12% SDS-PAGE in both reducing and non-reducing conditions. The antibodies were detected using Mouse anti-*myc* IgG and HRP-labeled goat anti-mouse IgG followed by chemiluminiscence detection. The figure depicts Western blot for 26434 and BR 2.3.

Based on binding assays and subsequent cloning into TG1 bacteria, 18 clones exhibiting the strongest binding specificities were selected and re-cloned into a suitable bacterial expression system for generating monomeric forms of sdAbs containing His- and *myc-*tags at the C-terminus for affinity purification and immuno-detection, respectively. For construction of pentamer or combody, the monomeric sdAb gene was fused with the N-terminus of COMP48 along with His- and *myc-*tags at the C-terminus. An illustration of the construction of the monomer and combody is shown in Figure 3A. Of the 18 clones generated, 16 were efficiently expressed in the bacterial periplasmic fraction and were purified using His-tag affinity columns (IMAC, GE Biosciences). The average yield of purified sdAbs ranged from 0.5 to 0.8 mg L^−1^ for both monomer and combodies. The purity of the protein was assessed by sodium-dodecyl sulfate polyacrylamide gel electrophoresis (SDS-PAGE) after purification.

The characterization of COMP48-conjugated pentameric sdAbs has been extensively discussed previously [32]. COMP is reported to exhibit inter-chain disulfide bonds at the C-terminus of the assembly domain. The pentamerization of 26424 and 26434 was confirmed by size-exclusion chromatography. Under non-reducing conditions, 26424 and 26434 migrated with a molecular weight of more than 130 kDa, suggesting that multimerization was attributed to inter-chain disulfide bonds (Figure 3B). Under reducing conditions, the 26424 and 26434 appeared as 25 kDa proteins (slightly higher molecular weight than the monomeric form due to the presence of linker sequences), confirming the presence of disulfide bonds (Figure 3C). The proteins were further analyzed by Western Blot using an anti-*myc* antibody and peroxidase-labeled anti-mouse IgG, followed by chemiluminescence detection (Figure 3D). By contrast, BR 2.3 appeared as 14 kDa monomer in SDS-PAGE under both reducing and non-reducing conditions and was further confirmed through size-exclusion chromatography (Sephadex 200, GE Healthcare), whereby monomeric sdAbs eluted quickly as a 14 kDa protein (Figure 3B and 3C).

### Specific recognition of sdAb monomer and combodies for RABV

**Figure 4:**
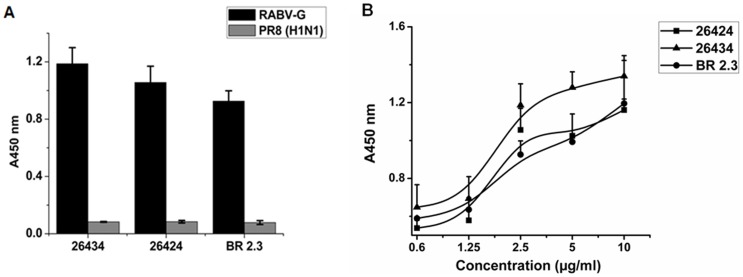
Binding of 26424, 26434 and BR 2.3 in ELISA to RABV. (A) The combodies, 26424 and 26434, and BR 2.3 (control monovalent sdAb) were analyzed in a binding ELISA to confirm their specificity to RABV (inactivated). PR8 (H1N1) was used as the negative control to determine cross-neutralization of the sdAbs. The graph depicts binding specificity of 26424, 26434, and BR 2.3 at a concentration of 2.5 μg ml^−1^. (B) Binding of 26424, 26434 and BR 2.3 to RABV at different concentrations of the purified sdAbs. Mouse anti-*myc* IgG was used as primary antibody followed by HRP-labeled goat anti-mouse IgG. The binding reactivity was confirmed in four independent experiments and the figure represents the average value. Mean ± standard deviations (SD) for each sample at different dilutions have been depicted.

The antigen-binding specificities of 26424, 26434 and BR 2.3, were determined via indirect enzyme-linked immunosorbent assay (Indirect ELISA) against immobilized whole RABV (inactivated) with influenza H1N1 virus (PR8) as a negative control. The binding of the sdAbs to the corresponding immobilized antigens were assessed using a mouse anti-*myc* mAb followed by anti-mouse horseradish peroxidase (HRP)–conjugated immunoglobulin (IgG). The purified combodies and monomer exhibited strong binding specificity to immobilized RABV, in contrast to a negligible or weak binding to H1N1 virus (PR8). Figure 4A depicts the binding specificities of 26424, 26434 and BR 2.3 to RABV at a median concentration of 2.5 μg ml^−1^. Binding specificity for both combodies and monomer was found to increase with higher concentrations of 26424, 26434 and BR 2.3 (Figure 4B).

### In vitro neutralizing efficacy of combodies against RABV pseudotypes

Neutralization abilities of 26424, 26434 and BR 2.3 were assessed in a standardized neutralization assay using the rabies CVS-11 pseudotype, a standard laboratory RABV strain. Pseudotypes or pseudoviruses, replaces the need for use of live viruses as they are antigenically similar to the native proteins on wild-type live viruses, and give high specificity as well as sensitivity for detection of virus neutralizing antibodies. Another advantage is that pseudotypes are replication-incompetent and can be used in biosafety level 2 (BL-2) laboratory conditions without the need for BL-4, which is essential in case of handling live pathogenic viruses.

**Figure 5:**
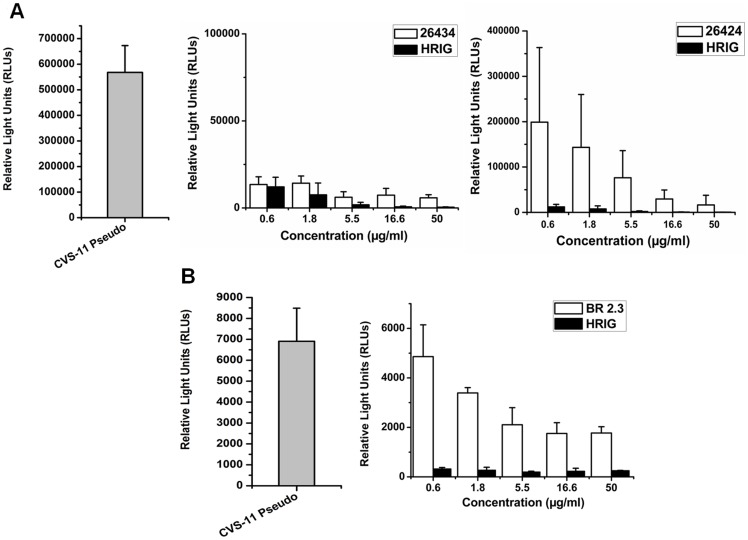
*In vitro* neutralization of CVS-11 pseudotypes by 26424, 26434 and BR 2.3. (A) BHK-21 cells were seeded into 96-well plate at 5×10^3^ cells per well along with pseudotypes (CVS-11 pseudo) with an input of 6×10^5^ RLUs. Wells treated with 26434 and 26424 as well as HRIG (positive control) showed inhibition of infection through decrease in RLUs, indicative of virus neutralization. The neutralization efficiencies with serial dilutions of 26424, 26434 and HRIG are depicted with reference to RLUs. (B) Neutralization assay of BR 2.3, a control sdAb in the monomer format isolated from the same naïve llama library. Lower titer of CVS-11 pseudotypes (7×10^3^ RLUs) was used to infect BHK-21 cells. Based on the ability to inhibit luciferase expression of the transduced cells, combodies 26424 and 26434 were able to neutralize 85-fold increased input of CVS-11 pseudotypes as compared to the monovalent BR 2.3. The relative neutralizing ability of 26424, 26434, and BR 2.3 were compared with similar concentrations of HRIG, currently used for post exposure prophylaxis (PEP) in rabies infection. All assays were carried out in triplicates and the graphs represent the average value. Standard deviations (± SD) are indicated by bars.

**Figure 6:**
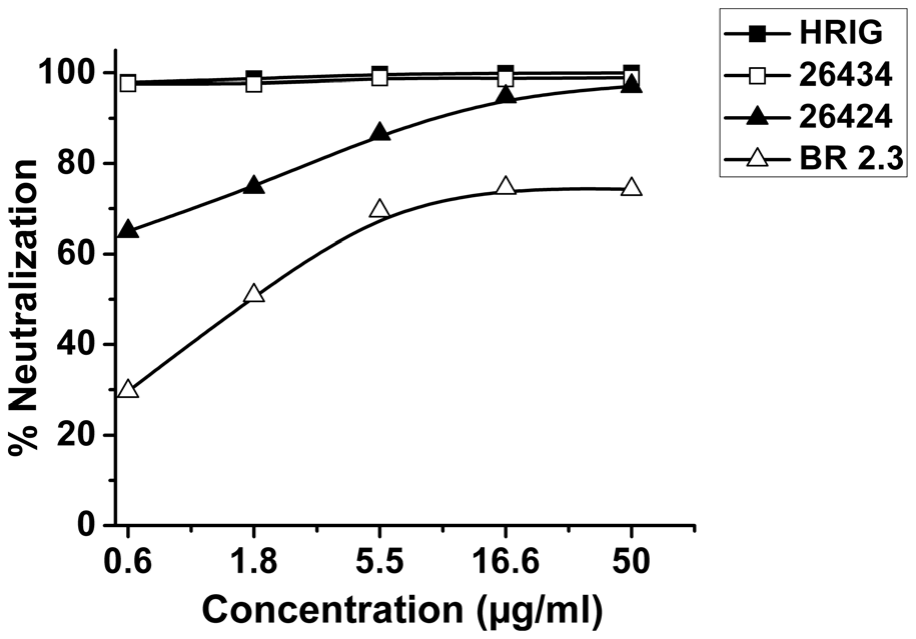
Percentage neutralization of 26424, 26434 and BR 2.3. Neutralization potencies have been calculated in percentage with reference to the decrease in RLUs of the antibody-treated samples as compared to negative control containing CVS-11 pseudotypes alone. Percentage neutralization of samples treated with 26424 and 26434 has been calculated against 6×10^5^ RLUs input of CVS-11 pseudotypes (Figure 5A), while that for BR 2.3 (control sdAb in monomer format) was calculated against 7×10^3^ RLUs of pseudovirus input (Figure 5B). HRIG was used as the positive control at similar concentrations of the test samples in all *in vitro* neutralization assays.

Pseudotypes for the CVS-11 strain were prepared by incorporating the envelope construct (G protein) of CVS-11 into a lentiviral vector (Invitrogen), which consists of the plasmids carrying the HIV *gag-pol* (pLP1) and the RSV promoter (pLP2). A firefly luciferase construct was used as a reporter for detection and analysis of neutralizing efficiencies of the sdAbs. Decrease in the relative light units (RLUs) of the luciferase activity in transduced BHK-21 cells was indicative of binding and neutralization of viral pseudotypes by the sdAb constructs. Combodies 26434 and 26424 could efficiently neutralize 85-fold higher input of CVS-11 pseudotypes (6×10^5^ RLUs) at a lower concentration of 5.5 μg ml^−1^ (Figure 5A). In contrast, however, at a relatively lower input range of CVS-11 pseudotypes (7×10^3^ RLUs), monovalent BR 2.3 was unable to inhibit infection of BHK-21 cells even at a much lower concentration (5.5 μg ml^−1^), but, could significantly neutralize CVS-11 pseudotypes at higher concentrations (Figure 5B). For 26424 and 26434, a dose of 16.6 μg ml^−1^ could neutralize 90% and 95% of the viral pseudotypes, respectively (Figure 6). HRIG, a standard positive control included in all the assays, could achieve 95–100% neutralization at similar range of concentrations. Furthermore, increase in the level of viral pseudotype titer was associated with concomitant decrease in the neutralizing ability of the sdAb constructs. This trend was found to be parallel with that of HRIG-treated samples. The data suggest that multimerization has contributed to the efficacies of 26434 and 26424 to be able to neutralize higher input of viral pseudotypes, resulting in increased neutralization potency *in vitro*.

### Protection of mice challenged by a lethal dose of RABV *in vivo* by combodies

**Figure 7:**
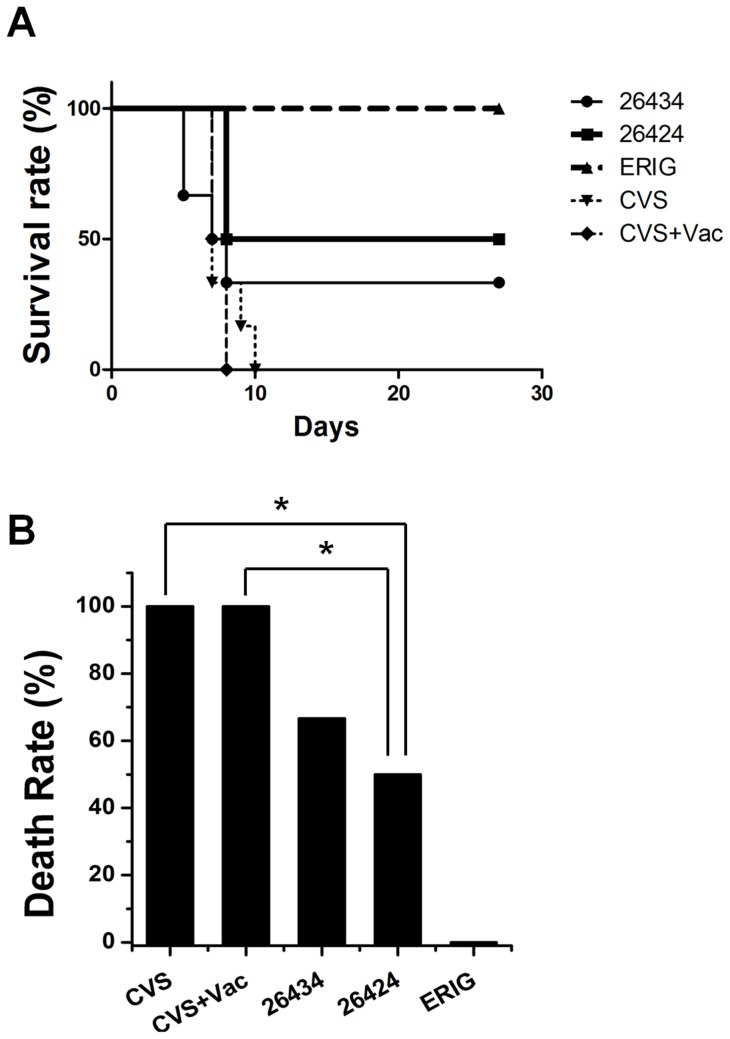
*In vivo* lethal challenge of rabies infection. (A) Kaplan–Meier survival curve for mice in post exposure prophylaxis with the sdAb pentamer constructs. Mice were challenged with 2500 LD_50_ CVS-24 strain of RABV mixed with 26424 (1.6 IU ml^−1^) and 26434 (0.2 IU ml^−1^) individually on Day 0. Negative control groups received PBS along with vaccine (CVS+Vac) or without vaccine (CVS) whereas positive control received 15.4 IU ml^−1^ equine rabies immunoglobulin (ERIG). Vaccination was done on day 0, 3, and 7 in all groups including negative and positive control. Animals were monitored daily for viability and weight change for a total of 28 days. Kaplan–Meier curves are shown by plotting percent survival against days (0 to 28). (B) Percentage death rate of each group after 28 days of observation. Mantel-Cox statistical analysis has been performed for all the groups. 26424 showed statistical significance in survival rate, compared to 26434 (* 0.01<P<0.05).

On the basis of their binding affinities to RABV in ELISA and effective neutralization of CVS-11 pseudotypes, the protection abilities of 26424 and 26434 for animals challenged by live rabies virus were tested in a mouse challenge model. The mouse neutralization test (MNT) was conducted under specific laboratory conditions to assess the protection of mice challenged by a lethal dose of RABV by the concurrent administration of the rabies vaccine together with 26424 and 26434 individually. ERIG, used as a positive control for MNT, is a cheaper and safe alternative to human RIG and is used for post exposure treatment of Rabies in developing countries. The survival rates of the mice during the 28-day observation period were plotted as Kaplan-Meier curves (Figure 7A) [42]. A death rate of 100% was observed in the negative control group receiving either CVS-24 virus only (CVS) or vaccine only (CVS+Vac), since the latter is not capable for immediate generation of neutralizing antibodies against RABV. The death rate in the group treated with 26424 (1.6 IU ml^−1^) was 50%, while the group treated with 26434 (0.2 IU ml^−1^) exhibited 60% death rate (Figure 7B). Positive control group, consisting of mice treated with vaccine and ERIG (15.4 IU ml^−1^), exhibited no death rate. The data implies that combodies, 26424 and 26434 were capable of neutralizing live RABV and could offer partial protection at a lower level of dosage. Taken together, the studies demonstrate that combodies, 26424 and 26434, could prove to be promising anti-viral molecules for Rabies infection *in vivo*.

## Discussion

In this study, we report the isolation of two neutralizing sdAbs, namely 26424 and 26434, from a naïve llama library against the trimeric glycoprotein (G) of RABV, and the influence of multimerization of the sdAbs to increase their neutralizing potential through oligomerization was investigated. The multimerization strategy for our study was adopted by fusing the coiled-coil peptide of the human cartilage oligomeric matrix protein 48 (COMP48) with sdAbs resulting in a pentavalent structure or combody. In contrast to single-chain variable fragments (scFvs), sdAbs are ideal candidates for oligomerization purposes, as they are half the size of scFvs and therefore produce smaller oligomeric forms. Furthermore, sdAbs can exist as monomers, whereas scFvs tend to form dimers, trimers, etc. [43]. COMP48 has been successfully used to generate high-avidity combodies specifically against the melanoma peptide-HLA A2 complex [32]. To our knowledge, this is the first attempt to evaluate the effect of multimerization of sdAb fragments using COMP48 for targeting antigens against infectious diseases such as Rabies.

In our experiments, the neutralizing potencies of combodies against RABV have been analyzed both *in vitro* and *in vivo*. Whole inactivated virus (aG strain) was used for selection, to obtain sdAbs specific for the RABV epitopes accessible in the intact viral particle. Initially, the binding properties of the sdAbs were evaluated using ELISA; the differences in the binding affinities of the various clones were presumed through initial rounds of bio-panning and phage ELISA. The clones exhibiting the strongest binding specificity were screened for further assessment of *in vitro* neutralizing ability using RABV (CVS-11) pseudotypes.

Several mechanisms might be responsible for the antiviral activities of the sdAbs. One possible mechanism is the blockade of the RABV-G protein interaction with its cognate cellular receptor, which thereby inhibits the virus to enter the cell and replicate. We have established a neutralization assay for testing the abilities of sdAbs to neutralize pseudoviruses *in vitro*. BHK-21 cells, which are routinely used in CVS-11 fluorescent antibody virus neutralization (FAVN) tests, are highly permissive for CVS-11 pseudotypes [44], [45]. Initially, 16 clones (data not shown), consisting of both monomers and combodies were tested in the neutralization assay, of which 26424 and 26434 could neutralize the CVS-11 pseudotypes with relatively high efficacy. The neutralizing abilities of the sdAbs have been compared with that of HRIG, currently used for post-exposure prophylaxis of rabies. As a proof-of-principle, further rabies pseudotype neutralization assays verified that the combodies, 26424 and 26434, could neutralize 85-fold increased input of CVS-11 pseudotypes *in vitro* at lower concentrations as compared to monovalent sdAb (BR 2.3), which highlights the improvement in avidity due to multimerization (Figure 5A, B and Figure 6).

It is to be noted that the sdAb genes were screened using the aG strain (also known as pG strain) of RABV as target antigen during bio-panning process. The G protein has eight amino substitutions (His_69_, Pro_184_, Pro_250_, Gly_427_, Ile_431_, Ile_477_, Lys_481_, and Asn_160_) which are unique to aG strain [33]. However, the antigenic sites, namely antigenic site I (231), antigenic site II (residues 34–42,198–200), antigenic site III (residues 330–338), antigenic site IV (residue 264) and antigenic site a (residue 342) were found to be conserved as compared to other vaccine strains [33]. *in vitro* assays of 26424 and 26434 (and also BR 2.3) against CVS-11 pseudotypes, suggest that the sdAb genes are specific for the G protein whose antigenic sites are conserved across wide variety of RABV strains. However, further investigations are needed for identifying the epitopes on the RABV G protein recognized by the combodies, in order to fully understand their future diagnostic or therapeutic value.

To investigate the neutralizing potencies of both the combodies, 26424 and 26434 against a lethal challenge *in vivo*, we performed the mouse neutralization test (MNT). The relative survival rate of mice treated with 26424 was approximately 50% compared to the 40% survival rate of mice that received 26434 (Figure 7). In the control groups, the mice receiving virus (CVS-24) alone or with vaccine exhibited 100% mortality within 10 days post-infection, whereas all of the ERIG-treated mice survived until day 28, post-infection or completion of the test. Combodies with a molecular weight of more than 130 kDa, sufficiently exceed the renal clearance threshold, could result in longer serum retention and produce effective viral neutralization. However, 26424 and 26434, could achieve partial protection (40–50%) as compared to 100% survival rate by ERIG. This may be partly due to the introduction of a human protein fragment (COMP48) with the sdAb gene that might elicit additional immunogenicity when injected in mice, resulting in decreased neutralization efficiencies *in vivo*. Moreover, as stated earlier, the relative concentration of 26434 (0.2 IU ml^−1^) and 26424 (1.6 IU ml^−1^) were lower than the standard dosage level required for effective virus neutralization *in vivo*. Our data comprises of preliminary investigations into the efficacy of multimeric sdAbs to be able to neutralize live RABV in mouse challenge model. Future work relating to dose-response studies is necessary to fully elucidate the prophylactic efficacies of 26434 and 26424 for achieving 100% protection in mice against rabies infection. Nonetheless, this study indicates that the neutralizing abilities of sdAbs have been significantly increased *in vitro* as well as *in vivo* as a result of multimerization.

We further addressed the issue of possible immunogenicity attributable to the repeated administration of non-human therapeutic proteins. The sdAbs are derived from *Camelidae* and exhibit significant homology to the human VH fragment. [46], [47], [48] The reduced immunogenic potential of the llama-derived heavy chain fragments (VHHs) have been further substantiated in primate studies performed by Ablynx (http://www.ablynx.com). On the other hand, the oligomeric matrix protein, COMP48, is of human origin, thus reducing the risk of immunogenicity upon administration in humans, despite possibly having elicited an immune response in the mouse neutralization test (MNT) as stated earlier. The shortcomings of such limitations might be negated, as long as the therapeutic protein is efficient in treating infectious diseases in humans. Furthermore, human-derived COMP48 has the added advantage of improved stability that results from complementary hydrophobic interactions and disulfide bridges between its α-helices.

In conclusion, multivalent sdAbs obtained through fusion with human COMP have been proven to exhibit increased avidity to target antigens. Moreover, fusion-antibodies exhibit a correct domain folding without compromising target specificity. The multivalent sdAbs isolated in our study could be useful anti-viral molecules for the treatment of RABV infection, as well as for investigations of mechanisms underlying viral infection, which remain poorly understood.
